# Motion-generation system for violin-playing robot using reinforcement learning differences in bowing parameters due to changes in learning conditions and sound pressure values

**DOI:** 10.3389/frobt.2024.1439629

**Published:** 2024-11-28

**Authors:** Kenzo Horigome, Koji Shibuya

**Affiliations:** ^1^ Mechanical Engineering and Robotics Course, Graduate School of Advanced Science and Technology, Ryukoku University, Otsu, Japan; ^2^ Mechanical Engineering and Robotics Course, Faculty of Advanced Science and Technology, Ryukoku University, Otsu, Japan

**Keywords:** reinforcement learning, humanoid, violin-playing robot, hidden layer, target sound pressure, total-reward

## Abstract

Recently, research on human-robot communication attracts many researchers. We believe that music is one of the important channel between human and robot, because it can convey emotional information. In this research, we focus on the violin performance by a robot. Building a system capable of determining performance from a musical score will leads to better understanding communication through music. In this study, we aim to develop a system that can automatically determine bowing parameters, such as bow speed and bowing direction, from musical scores for a violin-playing robot to produce expressive sounds using reinforcement learning. We adopted Q-learning and ε-greedy methods. In addition, we utilized a neural network to approximate the value function. Our system uses a musical score that incorporates the sound pressure value of each note to determine the bowing speed and direction. This study introduces the design of this system. It also presents simulation results on the differences in bowing parameters caused by changes in learning conditions and sound-pressure values. Regarding learning conditions, the learning rate, discount rate, search rate, and the number of units in the hidden layer in the neural network were changed in the simulation. We used the last two bars of the score and the entire four bars in the first phrase of “Go Tell Aunt Rhody.” We determined the number of units in each layer and conducted simulations. Additionally, we conducted an analysis by adjusting the target sound pressure for each note in the score. As a result, negative rewards decreased and positive rewards increased. Consequently, even with changes in target sound pressure in both the last two bars and the entire four bars, the violin-playing robot can automatically play from the score by improving reinforcement learning. It has become clear that achieving an expressive performance using this method is possible.

## 1 Introduction

### 1.1 Unique trends in this research

Communications research has become increasingly active worldwide in recent years. Robots are being developed and used to enable communication between humans and other robots. Examples include industrial robots working in factories, nursing care robots working in hospitals and elderly care facilities, robots that communicate directly with humans through voice, and robots that play musical instruments. These robots can move in ways similar to humans and animals, performing tasks that humans cannot do. Consequently, they have the potential to collaborate and communicate with humans, supporting and enriching people’s lives in various ways.

Research on robots playing musical instruments has been ongoing for a long time. For example, there are organ-playing robots by Kato et al. (1987), MUBOT by Kajitani (1989), guitar-playing robots (Sobh et al., 2003), bagpipe-playing robots (Solis and Ng, 2011), marimba-playing robots (Bretan and Weinberg, 2019), performance robots (Petersen et al., 2010), and saxophone performance robots (Lin et al., 2019), which have become popular. In addition, organ-playing robots (Sugano et al., 1986) have been studied using algorithms that automatically determine performance movements based on musical scores. This study focuses on violins, whose sounds are determined by bowing.

Regarding violin-playing robots, (Kuwabara et al., 2006), and (Min et al., 2013) developed mechanisms and control systems for robot arms and hands. In addition, Toyota Motor Corporation developed a humanoid violin-playing robot (Kusuda, 2008), but no academic research has been conducted on it. This study aims to construct a process for determining playing movements from musical scores as academic research using a violin-playing robot that operates with two humanoid arms (Shibuya et al., 2020a).

Shibuya et al. (2020b) constructed an algorithm to determine the performance motion based on data obtained by analyzing the parameters of bow movement when producing a tone based on the performance of a human violinist. However, this approach applies only to a specific piece of music and not to various pieces, necessitating changes in performance design each time the piece of music changes. Therefore, to improve efficiency, we aim to generate performance sounds according to the target sound pressure using a value function approximation with a reinforcement learning neural network and automate bowing decisions (Shibuya et al., 2020a; Shibuya et al., 2022).

In Shibuya et al. (2020a), only the discount rate γ was considered, whereas in Shibuya et al. (2022), both the discount rate γ and the search rate ε were considered. However, in both studies (Shibuya et al., 2020a; Shibuya et al., 2022b), differences in bowing parameters owing to changes in the maximum and minimum values of the reward, the number of bars in the score, number of units in the hidden layer (middle layer), and target sound pressure value were not considered. Therefore, in this study, we used the analysis results to examine the differences in bowing parameters owing to changes in the maximum and minimum reward values, number of bars in the score, number of units in the hidden layer (middle layer), and target sound pressure value. This approach aimed to address these variations effectively.

### 1.2 Violin-playing robot

**FIGURE 1:**
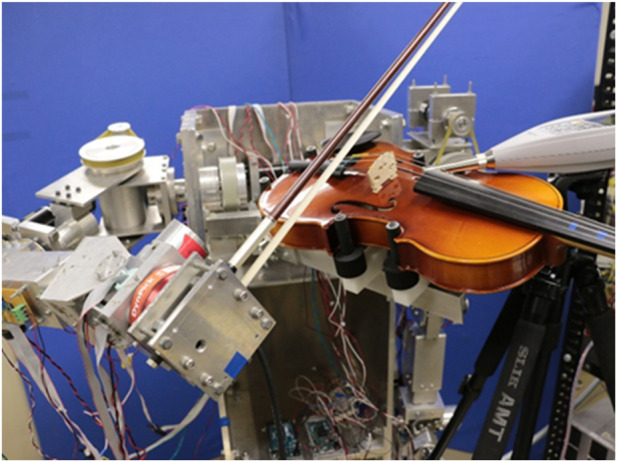
Violin-playing robot.

Figure 1 shows the violin-playing robot that is the subject of this study.

**FIGURE 2:**
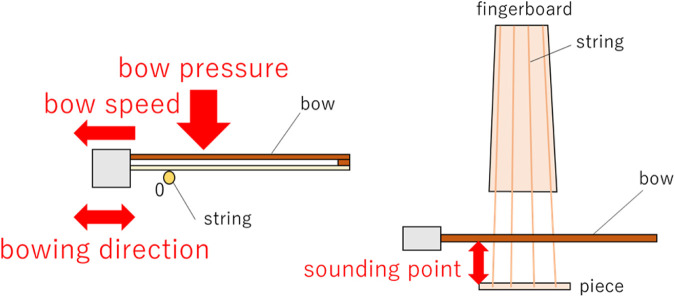
Parameters of right arm bowing in violin performance.

This robot is a humanoid dual-armed robot that has joints with 7 degrees of freedom in both arms and is driven by DC motors. The violin performance of this robot consists of bowing with the right arm, fingering with the left hand, and holding the instrument. Furthermore, the bow movement of the right arm can be divided into bow speed, bow pressure, sounding point, and direction of bow movement, as shown in Figure 2.

### 1.3 Performance movement plan

It is possible to design the bow speed and bowing direction according to the designated sound pressure without reinforcement learning. However, when including other parameters such as the bow force, fingering, or use of different musical pieces, the complexity of the performance design is greatly increase. In our system, we used reinforcement learning to avoid such issues.

We aim to develop a robot that can automatically determine performance actions based on the piece being played, similar to a human violinist, to achieve an expressive performance. Moreover, we aim to design a robots that can effectively communicate with people through music.

Regarding the motion generation system and its determination of performance motions, we discuss how generated performances differ depending on performance skill, the impact on the sound and audience, and the ability to perform musically regardless of circumstances. These decisions are made from the perspective of the specialized content of mechanical systems engineering.

From the perspective of playing techniques, the main actions are “bowing with the right arm” and “fingering with the left hand.” The ability to adjust bow speed (the speed at which the bow is played) and bow pressure (the force applied to the bow string) varies depending on the performer. In addition, during left-hand fingering, the ability to adjust the force of the fingers when pressing the strings and the accuracy of the pitch also differ depending on the performer. Based on this premise, when generating motions for a short and simple musical score, we can determine how different the performance motions are depending on the bowing and fingering conditions and whether the performance is musical based on the evaluation of the performance sound.

Given that the main body of the violin-playing robot, shown in Figure 1, has been completed as hardware, future work will adopt reinforcement learning to quickly respond to specified sound conditions by adjusting the strength, volume, and other characteristics of the sound. We will develop a system that determines the movement of the bow using the robot’s right arm and improve the system so that the robot can automatically determine the performance motion from the musical score. Simply improving the robot’s hardware has its limitations in determining the robot’s performance movements from musical score data. Therefore, reinforcement learning (a part of machine learning) has been adopted to maximize the total reward.

Rhythm, tone, and note length are also important parameters that should be considered. However, in this study, we focus only on sound pressure for the sake of simplicity. We hypothesize that by following the sound pressure levels of a human violinist, a more human-like performance will be achieved.

## 2 Methods

### 2.1 General explanation of Q-learning

In this section, we briefly explain the concept of Q-learning. In the following discussion, let t be the episode (number of trials). First, the agent (machine itself) receives the state $S_{t}$ , information about the environment (surroundings), and generates the existing value V . Immediately after the agent performs action $a_{t}$ , which is decided by the agent based on the existing value V , the agent obtains reward $r_{t+1}$ , thereby updating the new value V from the existing value V . This task is repeated for the designated number of trials while maximizing the cumulative reward (total reward). Based on this framework, the Q value is calculated according to Equation 1.

$$Q\left(S_{t},a_{t}\right)=Q\left(S_{t},a_{t}\right)+\alpha \left(r_{t+1}+\gamma \cdot maxQ\left(S_{t+1},a_{t+1}\right)-Q\left(S_{t},a_{t}\right)\right)$$ (1)

Here, $Q\left(S_{t},a_{t}\right)$ calculates the Q value, which is used to measure the optimality of the action selection of the robot; α is the learning rate, which is used to adjust the learning speed; and γ is the discount rate, which is used to determine the present value of future rewards. In addition, the ε -greedy method is used as a strategy to obtain the Q value. The ε -greedy method is used to randomly select an action from all actions with probability 1−ε and select a high-value action with the remaining probability ε . The larger the probability ε , the higher the probability that the machine itself will explore an action. Conversely, the smaller the probability ε , the higher the probability that the machine itself will increase the value of the action. In Equation 1, the sum of the product of the maximum state-action value of the next episode and the discount rate is determined, and the reward is defined as the revenue. As the calculation progresses, the error between the revenue and the state-action value of the previous episode approaches zero. The learning momentum is measured using the learning rate, and the state-action value is updated.

### 2.2 Reinforcement learning for a violin-playing robot

Unlike supervised and unsupervised learning, reinforcement learning does not require data to be input to the robot, and the robot can output data by taking an action while automatically acquiring data. Q-learning is a form of reinforcement learning, and unlike dynamic programming, it does not require a complete model with prior knowledge of the environment. Learning can proceed by approximating the value function via experience gained from state-action interactions. In addition, unlike the actor-critic method, it can update the value of both the state and action simultaneously. Furthermore, unlike the Sarsa method, it can select the action with the greatest value in the next state. Therefore, we used Q-learning in the proposed system.

**FIGURE 3:**
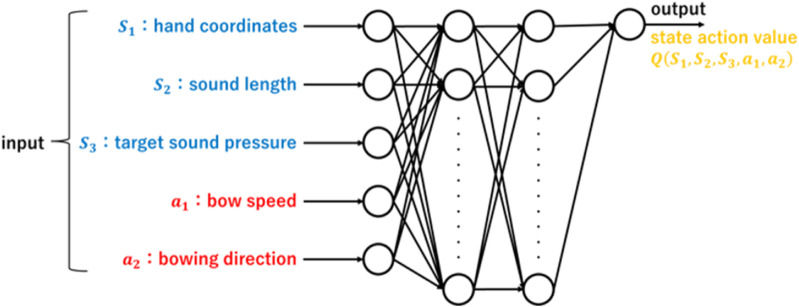
Value function approximation in reinforcement learning neural networks.

In this study, we used reinforcement learning to determine the performance motions of a violin-playing robot. For state S , the coordinates of the hand were set to $S_{1}$ [mm], sound length was set to $S_{2}$ [s], and target sound pressure was set to $S_{3}$ [dB]. For action a, the bow speed was set to $a_{1}$ [mm/s], and the direction of the bow movement was set to $a_{2}$ . As shown in Figure 3, we adopted Q-learning and ε-greedy method. The state *S* and action *a* were the input, the value function approximation was performed by Q-learning in a neural network via a hidden layer, and the Q-value was the output. Although a simple state-space model can be used, if the score becomes long, the number of Q values that must be set increases. Therefore, in this study, we decided to employ a value function approximation using a neural network.

To determine the bow speed such that the output sound pressure *Vo* approaches the target sound pressure *Vi*, it is necessary to derive the relationship between the output sound pressure and bow speed. This relationship is given by Equation 2, where $V_{o}$ [dB] is the output sound pressure and v [mm/s] is the bow speed.

$$V_{o}=-0.0015v^{2}+0.3286v+57.704$$ (2)

In addition, the reward is given by Equation 3, where r is the reward, k is a constant, $V_{i}$ [dB] is the target sound pressure, and $V_{o}$ [dB] is the output sound pressure.

$$r=\frac{k}{\;\left|V_{i}-V_{o}\right|\;}$$ (3)

Here, the value of the constant k is set to 1. If the bow motion generation is successful, the upper limit of the reward r for one note is set to 1. If the bow motion generation fails, r is set to −1, regardless of Equation 3.

**FIGURE 4:**
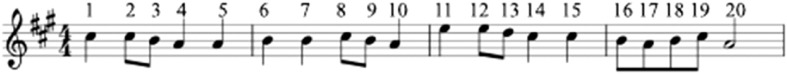
Musical score used for analysis.

We created a program code in C++ for the last two measures and all four measures of the musical score shown in Figure 4, and reinforcement learning analysis was performed with 300 performance trials. The numbers 1 to 20 shown in Figure 4 are assigned to each note in order from left to right.

In the reinforcement learning parameters, we set the search rate ε to 0.05, learning rate α to 0.5, and discount rate γ to 0.5. In previous research, which used only one hidden layer, the number of units in the hidden layer was set to 15. In this study, two hidden layers were used, with the number of units $h_{1}$ in the first hidden layer and $h_{2}$ in the second hidden layer changing based on Table 1.

**TABLE 1:** Number of units in the hidden layer.

Order	1	2	3	4	5	6
$h_{1}$	10	10	10	30	30	30
$h_{2}$	5	10	15	25	30	35

As the number of hidden layers and their units increases, the calculation time required to approximate the value function increases significantly, and the time required for learning increases accordingly. In some cases, the computational load may make learning impossible. Therefore, the number of hidden layers was set to two, and the number of units was limited to a maximum of 35.

We next consider the number of units. Specifically, when only one hidden layer was used and the number of units was varied, it was determined that 10 or 30 units is suitable for learning. Based on this finding, the number of units used for two hidden layers was set as shown in Table 1.

For the target sound pressure of each note in the musical score shown in Figure 4, previous research set sound pressure values based on measurements from actual human violin performances. We determined the target sound pressure level based on the performance experiment reported in (Shibuya et al., 2020a), in which a music college student majoring in violin participated. We subtracted 11 dB from the human data to ensure that the robot produced the target sound pressure. In this study, the sound pressure was set to increase in the first half of the song and decrease in the second half. This pattern is one of many variations in sound pressure. Table 2 presents the target sound pressure of each note in the previous and present studies.

**TABLE 2:** Target sound pressure values used for analysis.

Note number	Previous research [dB]	This research [dB]
1	67.74	66.74
2	73.14	67.00
3	76.00	67.14
4	68.78	67.78
5	66.97	67.97
6	74.77	68.36
7	75.36	68.77
8	69.04	68.99
9	75.99	69.30
10	71.30	70.04
11	72.06	70.06
12	66.54	70.54
13	73.56	71.56
14	74.00	71.00
15	72.60	70.00
16	69.87	69.87
17	72.00	68.00
18	67.79	67.79
19	73.97	66.97
20	66.56	66.56

## 3 Results

First, data were obtained by changing $h_{1}$ to the number of units in the first hidden layer and $h_{2}$ to the number of units in the second hidden layer, in the order presented in Table 1, targeting the latter two measures of the score, as shown in Figure 4. From this, we calculated the learning success rate and maximum positive reward, as listed in Table 3. The learning success rate was calculated as the percentage of positive reward values out of 300 reward values. In addition, the maximum positive reward value was calculated using the highest positive reward value among the 300 reward values.

**TABLE 3:** Learning success rate and maximum positive reward in the latter two measures of the score and two hidden layers.

$h_{1}$	$h_{2}$	Success rate [%]	Maximum positive reward
10	5	60.0	7.41
10	10	73.7	8.03
10	15	85.3	7.69
30	25	71.7	7.60
30	30	85.3	8.12
30	35	82.3	8.17

From Table 3, it can be observed that the learning success rate exceeds 80% when $h_{1}$ = 10 and $h_{2}$ = 15, $h_{1}$ = 30 and $h_{2}$ = 30, and $h_{1}$ = 30 and $h_{2}$ = 35. Moreover, the learning success rate is highest when $h_{1\;}$ = 10 and $h_{2}$ = 15 and $h_{1\;}$ = 30 and $h_{2}$ = 30, and the maximum positive reward is highest when $h_{1}$ = 30 and $h_{2}$ = 35.

**FIGURE 5:**
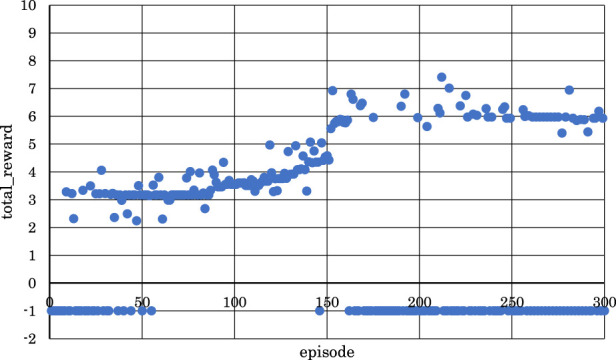
Correlation between episodes and total rewards when $h_{1}$ = 10 and $h_{2}$ = 5 (2 bars).

**FIGURE 6:**
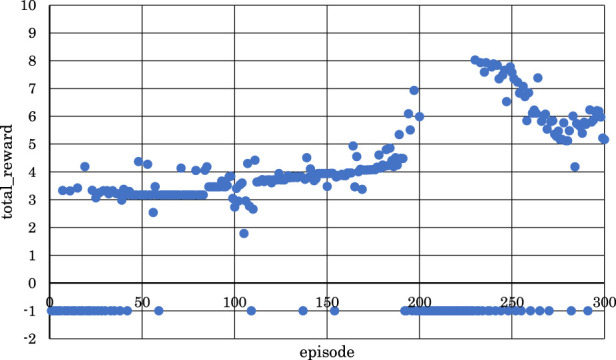
Correlation between episodes and total rewards when $h_{1}$ = 10 and $h_{2}$ = 10 (2 bars).

**FIGURE 7:**
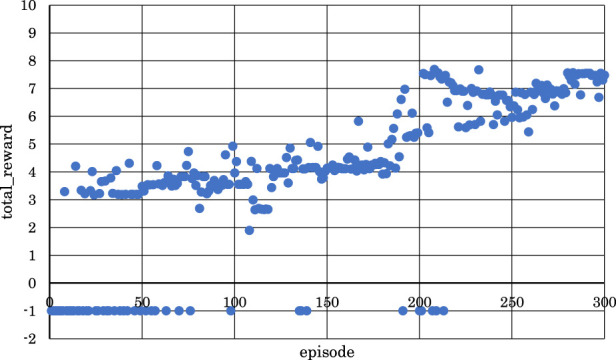
Correlation between episodes and total rewards when $h_{1}$ = 10 and $h_{2}$ = 15 (2 bars).

**FIGURE 8:**
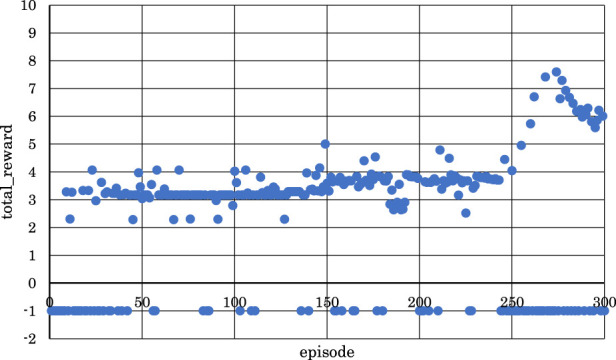
Correlation between episodes and total rewards when $h_{1}$ = 30 and $h_{2}$ = 25 (2 bars).

**FIGURE 9:**
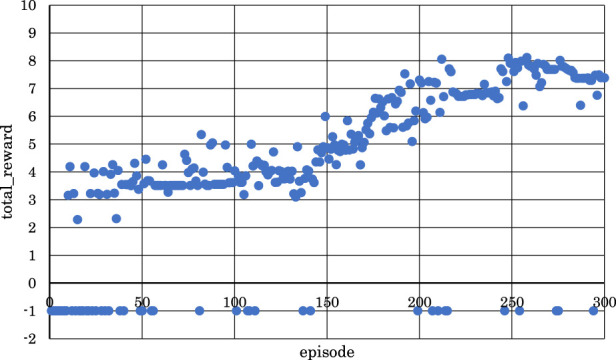
Correlation between episodes and total rewards when $h_{1}$ = 30 and $h_{2}$ = 30 (2 bars).

**FIGURE 10:**
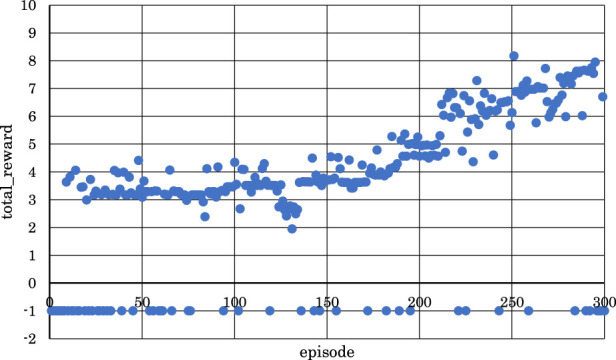
Correlation between episodes and total rewards when $h_{1\;}$ = 30 and $h_{2}$ = 35 (2 bars).

Figures 5–10 show the correlation between the episodes (number of performance trials) and total rewards (sum of rewards for each note in one episode).

From Figures 5, 6, 8, where $h_{1}$ = 10 and $h_{2}$ = 5, ${\;h}_{1}$ = 10 and $h_{2}$ = 10, or $h_{1}$ = 30 and $h_{2\;}$ = 25, it can be observed that in the latter half of learning, the negative rewards representing failure continued while the positive rewards representing success decreased and were minimized.

Conversely, from Figures 7, 9, 10, where $h_{1}$ = 10 and $h_{2\;}$ = 15, $h_{1}$ = 30 and $h_{2\;}$ = 30, or $h_{1\;}$ = 30 and $h_{2}$ = 35, it can be observed that as the number of episodes increased, negative rewards representing failure decreased while positive rewards representing success increased and were maximized.

From the above, it became clear that, reinforcement learning is effective when $h_{1\;}$ = 10 and $h_{2}$ = 15, $h_{1}$ = 30 and $h_{2\;}$ = 30, or $h_{1\;}$ = 30 and $h_{2}$ = 35. In other words, it became clear that, as the violin-playing robot succeeded in generating the bow motion, it was able to bring the output sound pressure closer to the target sound pressure and play the violin according to the target sound pressure.

Next, based on the results in Table 3 and Figures 5–10, data were obtained by changing $h_{1}$ to the number of units in the first hidden layer and $h_{2}$ to the number of units in the second hidden layer only in the order of 3, 5, and 6 in Table 1, targeting the entire four measures of the score shown in Figure 4. From this, we calculated the learning success rate and maximum positive reward, as summarized in Table 4.

**TABLE 4:** Learning success rate and maximum positive reward in the entire four measures of the score and two hidden layers.

$h_{1}$	$h_{2}$	Success rate [%]	Maximum positive reward
10	15	70.3	15.9
30	30	76.7	16.0
30	35	71.3	15.7

From Table 4, you can observe that the highest values for both the learning success rate and the maximum positive reward were achieved when $h_{1}$ = 30 and $h_{2}$ = 30. This indicates that throughout the 300 episodes, the negative rewards representing failure were minimized while the positive rewards representing success were maximized. Simultaneously, the maximum positive reward representing success reached its highest value.

**FIGURE 11:**
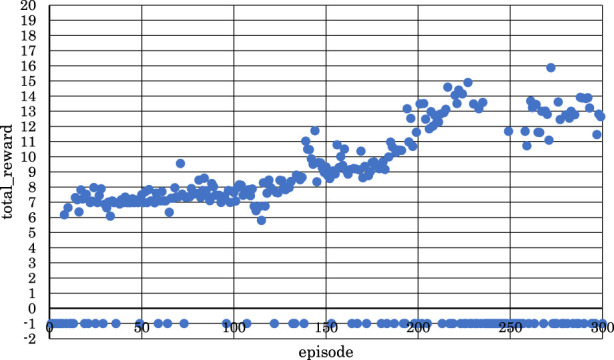
Correlation between episodes and total rewards when $h_{1\;}$ = 10 and $h_{2}$ = 15 (4 bars).

**FIGURE 12:**
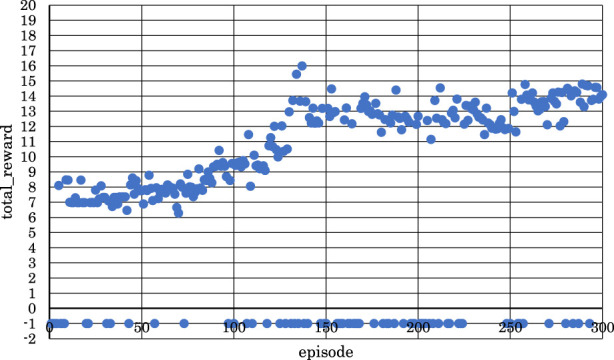
Correlation between episodes and total rewards when $h_{1}$ = 30 and $h_{2\;}$ = 30 (4 bars).

**FIGURE 13:**
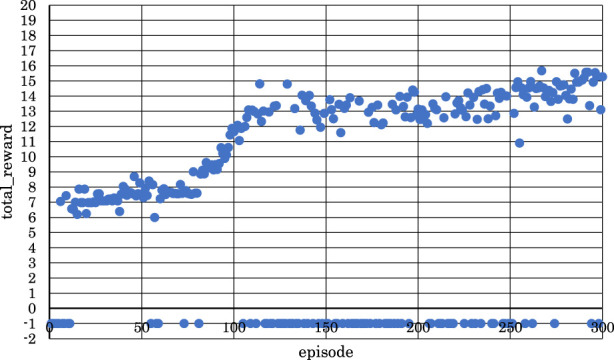
Correlation between episodes and total rewards when $h_{1}$ = 30 and $h_{2}$ = 35 (4 bars).

Figures 11–13 show the correlation between the episodes (number of performance trials) and total rewards (sum of rewards for each note in one episode).

From Figure 11, we can observe that the positive rewards, representing success, decreased in the latter half of the learning process when $h_{1}$ the number of units in the first hidden layer, and $h_{2},$ the number of units in the second hidden layer, were set to $h_{1}$ = 10 and $h_{2}$ = 15.

From Figure 12, it is evident that the positive rewards increased, and the range of increase was wide in the early stages of learning when $h_{1}$ and $h_{2}$ were set to $h_{1\;}$ = 30 and $h_{2\;}$ = 30.

From Figure 13, we can see that the positive rewards also increased, but the range of increase was narrower in the early stages of learning when $h_{1}$ and $h_{2}$ were set to $h_{1\;}$ = 30 and $h_{2}$ = 35.

From these observations, it became clear that reinforcement learning is effective in both the latter two measures and the entire four measures of the musical score shown in Figure 4 when $h_{1}$ and $h_{2}$ were both set to 30. This means that as the violin-playing robot succeeded in generating the bow motion, it was able to bring the output sound pressure closer to the target sound pressure and play the violin according to the target sound pressure.

**FIGURE 14:**
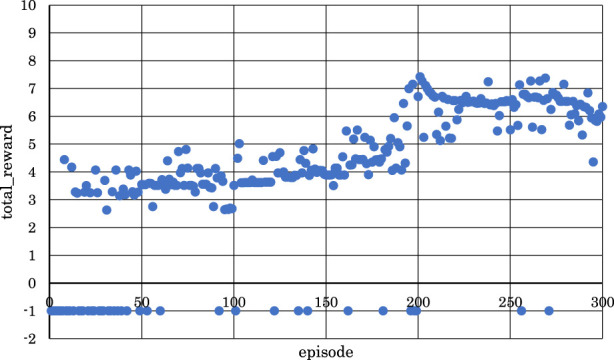
Correlation between episodes and total rewards after changing target sound pressure (2 bars).

**FIGURE 15:**
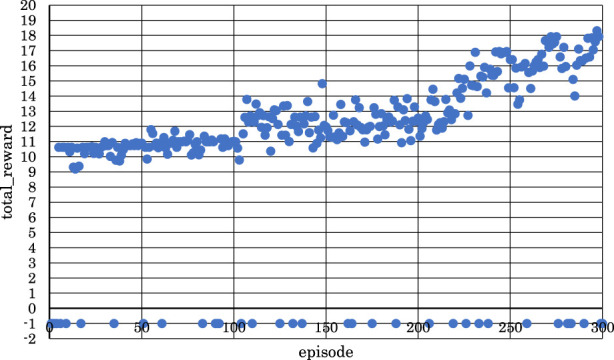
Correlation between episodes and total rewards after changing target sound pressure (4 bars).

Finally, with both $h_{1}$ and $h_{2}$ set to 30, we adjusted the target sound pressure values for each note according to Table 2 for both the latter two measures and the entire four measures of the musical score shown in Figure 4. Figures 14, 15 show the correlation between the episodes (number of performance trials) and the cumulative reward (sum of rewards for each note in one episode) after changing the target sound pressure values.

As shown in Figure 14, as the number of episodes increased, the negative rewards representing failure gradually decreased, and the positive rewards representing success gradually increased. Similarly, as shown in Figure 15, as the number of episodes increased, the negative rewards representing failure were not continuous, and overall, the positive rewards representing success gradually increased.

In Figure 14, the learning success rate was 86.3%, and the maximum positive reward was 7.43. In addition, as shown in Figure 15, the learning success rate was 88.0%, and the maximum positive reward was 18.3.

From the above findings, it is clear that reinforcement learning was effective even when the number of units in each layer was changed, and the numerical value of the target sound pressure of each note in the score was changed for both the latter two measures and the entire four measures of the musical score, as shown in Figure 4. In other words, it was evident that, as the violin-playing robot succeeded in generating the bow motion, it was able to bring the output sound pressure closer to the target sound pressure and play the violin according to the target sound pressure.

It should be noted that increasing the number of hidden units does not necessarily result in a higher success rate. Therefore, the number of hidden units used in this study was determined to be appropriate, although the understanding of this relationship remains unclear.

## 4 Conclusion

**FIGURE 16:**
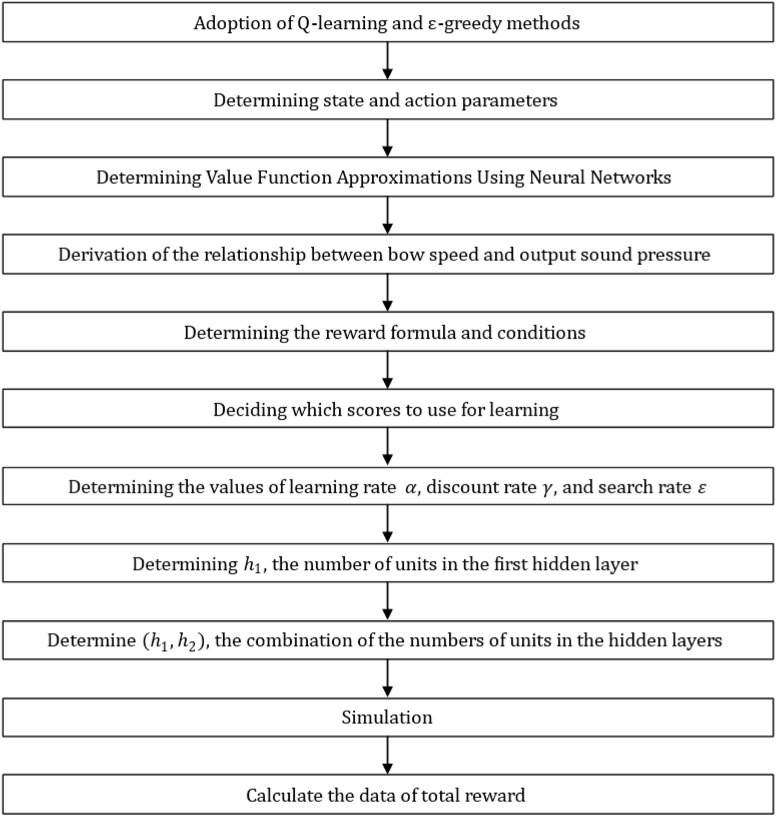
Work flowchart.

**FIGURE 17:**
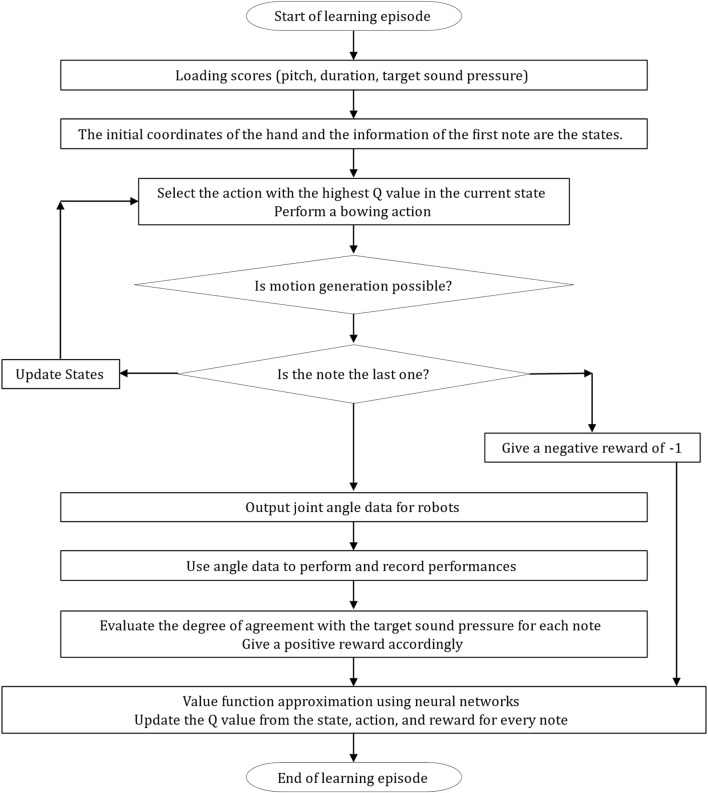
Flowchart for the C++ coding of the learning episode.

In this study, we examined the number of hidden layers, number of units in each layer, and pattern of sound pressure changes in a piece of music with the aim of enabling a violin-playing robot to automate its playing movements based on musical scores. Using reinforcement learning (Q-learning), we generated a neural network and performed value-function approximation on the network. We carried out the analysis according to the order in Figure 16 and the flowchart in Figure 17.

However, from a musical perspective, the discussion above is limited to the scores shown in Figure 4. Therefore, in the future, we will apply other musical scores (music pieces) in addition to those shown in Figure 4, specify the bar range of the musical score to be subjected to reinforcement learning, perform reinforcement learning analysis (including simulations), and examine changes in the correlation between episodes (number of performance trials) and the total reward (sum of rewards for each note in one episode).

In addition, from the perspective of performance movement when playing the violin, the bow movement of the right arm of the violin-playing robot shown in Figure 1 is limited by the bow speed and direction of bow movement. Therefore, in the future, we will perform reinforcement learning analysis (simulation) that considers not only the bow pressure and sounding point of the robot’s right arm bowing movement but also the fingering of the robot’s left hand. It is necessary to examine the changes in the correlation between episodes (number of performance trials) and total rewards (sum of rewards for each note in one episode).

Furthermore, from the perspective of musical performance planning, the discussion has been limited to the analysis (simulation) of reinforcement learning, and it remains unclear whether reinforcement learning is possible when operating a violin-playing robot on an actual machine.

For example, when generating sounds of the same pitch, there are multiple ways to move the bow, such as “up bow” and “down bow.” The question is whether a violin-playing robot can simultaneously perform bow movement and sound generation, automatically determine the playing motion from the musical score, and play the violin as analyzed.

Therefore, in the future, it will be necessary to operate a violin-playing robot on an actual machine to perform reinforcement learning.

## Data Availability

The original contributions presented in the study are included in the article/supplementary material, further inquiries can be directed to the corresponding author.
